# Germanium Dioxide Nanoparticles Mitigate Biochemical and Molecular Changes Characterizing Alzheimer’s Disease in Rats

**DOI:** 10.3390/pharmaceutics15051386

**Published:** 2023-04-30

**Authors:** Sara A. Abdel Gaber, Amal H. Hamza, Mohamed A. Tantawy, Eman A. Toraih, Hanaa H. Ahmed

**Affiliations:** 1 Nanomedicine Department, Institute of Nanoscience and Nanotechnology, Kafrelsheikh University, Kafr El Sheikh 33516, Egypt; 2 Biochemistry and Nutrition Department, Faculty of Women, Ain Shams University, Cairo 11566, Egypt; 3 Hormones Department, Medical Research and Clinical Studies Institute, National Research Center, Dokki, Giza 12622, Egypt; 4 Stem Cell Lab, left of Excellence for Advanced Sciences, National Research Centre, Dokki, Giza 12622, Egypt; 5 Genetics Unit, Histology and Cell Biology Department, Faculty of Medicine, Suez Canal University, Ismailia 41522, Egypt

**Keywords:** Alzheimer’s disease, germanium dioxide nanoparticles, cerium dioxide nanoparticles, MicroRNAs, antioxidant, Alzheimer´s biomarkers

## Abstract

Alzheimer’s disease (AD) is a neurodegenerative disorder that jeopardizes the lives of diagnosed patients at late stages. This study aimed to assess, for the first time, the efficiency of germanium dioxide nanoparticles (GeO_2_NPs) in mitigating AD at the in vivo level compared to cerium dioxide nanoparticles (CeO_2_NPs). Nanoparticles were synthesized using the co-precipitation method. Their antioxidant activity was tested. For the bio-assessment, rats were randomly assigned into four groups: AD + GeO_2_NPs, AD + CeO_2_NPs, AD, and control. Serum and brain tau protein, phosphorylated tau, neurogranin, amyloid β peptide 1-42, acetylcholinesterase, and monoamine oxidase levels were measured. Brain histopathological evaluation was conducted. Furthermore, nine AD-related microRNAs were quantified. Nanoparticles were spherical with diameters ranging from 12–27 nm. GeO_2_NPs exhibited a stronger antioxidant activity than CeO_2_NPs. Serum and tissue analyses revealed the regression of AD biomarkers to almost control values upon treatment using GeO_2_NPs. Histopathological observations strongly supported the biochemical outcomes. Then, miR-29a-3p was down-regulated in the GeO_2_NPs-treated group. This pre-clinical study substantiated the scientific evidence favoring the pharmacological application of GeO_2_NPs and CeO_2_NPs in AD treatment. Our study is the first report on the efficiency of GeO_2_NPs in managing AD. Further studies are needed to fully understand their mechanism of action.

## 1. Introduction

Alzheimer’s disease (AD) is one of the most common neurodegenerative illnesses, resulting in gradual dementia in the elderly population [1]. It represents the fifth-leading cause of mortality, with a prevalence of more than 46 million globally, according to AD International Recordings [2]. Although the specific causation is still unknown, many validated studies proved that AD is caused by a mix of hereditary and environmental factors. Several previous studies indicate that the accumulation of toxic proteins, specifically tau tangles and amyloid β peptide (Aβ) plaques, and their movement in and out of brain neurons, serve as primary mechanisms for the onset of AD [3]. Thus, AD is distinguished by intracellular neurofibrillary tangles (NFTs) and extracellular senile Aβ plaques in the brain. NFTs are abnormally hyperphosphorylated microtubules linked together by the tau protein [4]. Only the Aβ cascade and tau hyperphosphorylation hypotheses have gained prevalent approval for the diagnosis of AD [5].

Promising studies showed the role of small non-coding microRNAs as diagnostic, prognostic, and predictive biomarkers in AD and miRNA combinations increase the sensitivity and specificity compared with single miRNAs [6]. Thus, miRNAs are particularly abundant in the neurological system, where they perform a role in neuronal activities, such as neurite outgrowth, dendritic spine architecture, neuronal development, and synaptic plasticity [7]. Of these, cerebrospinal fluid (CSF) miR-125b-5p, miR-146a-5p, and miR-29a-3p levels were a better ancillary panel to Aβ 1-42: Total-tau ratio that differentiates between AD and controls [6].

A panel of 10 microRNAs, including miR-107, miR-146a, and miR-125b, was deregulated early in AD, nearly 20 years before the onset of clinical symptoms [8]. Dysregulation of miR-9a-5p was found to be associated with altered brain-enriched gene expression patterns that, in part, characterize AD neuropathology [9]. Some miRNAs have steadily been deregulated in cellular and animal AD models. It has been shown that voluntary physical exercise modulates the expression of several miRNAs involved in AD, such as miR-30a-5p and miR-128, in a spontaneous senescence-accelerated P8 (SAMP8) mouse model [10]. Down-regulation of miR-181c-5p in the hippocampus of SAMP8 mice is also known to perform a role in AD pathogenesis [11]. These microRNAs can be used as candidate biomarkers in clinical practice, assisting in identifying novel proteins and pathways linked to AD, and they may reveal novel future therapeutic targets for AD. Currently, the treatment modalities aim to relieve the symptoms and delay the disease progression. The available medications authorized by the FDA are cholinesterase inhibitors, which raise acetylcholine concentrations and, thereby, neurotransmitter activity [12].

Alternative and cutting-edge treatments, such as nanomedicine, have been suggested for AD. In this regard, nanoparticles displaying free radical scavenging properties and antioxidant activity, such as cerium dioxide nanoparticles (CeO_2_NPs), are of great interest. Several studies have reported that CeO_2_NPs are free radical scavengers in vitro and in vivo [13]. CeO_2_NPs shuttle between their 3þ and 4þ states, and oxidation of Ce4þ to Ce3þ causes oxygen niches and defects on the surface of the crystalline lattice structure, producing a circumstance for the occurrence of redox reactions. As a result, CeO_2_NPs mimic the catalytic activities of several antioxidant enzymes [14]. Additionally, CeO_2_NPs are widely used in treating medical disorders caused by reactive oxygen intermediates due to their unique redox properties [15].

Germanium is a naturally occurring nonessential element. The biomedical implementations of germanium are so limited [16], but it has been shown that germanium has anti-cancer and anti-viral properties in vivo and antitumor, anti-aging, and anti-inflammatory properties in vivo and in vitro. Furthermore, organic germanium compounds effectively improve immune function in different pathological conditions in preclinical studies [17]. In addition, germanium is effective against liver diseases, hypertension, arthritis, food allergies, autoimmune diseases, senile osteoporosis, and malaria [16]. Various enzymes have been reported to be inhibited by germanium, including alcohol dehydrogenase, glutamic oxaloacetic transaminase, glutathione-S-transferase, and lactic dehydrogenase [18]. Germanium dioxide nanoparticles (GeO_2_NPs) became the focus of many studies that aimed to control their shape [19] and synthesize them in reverse micelles [20]. Their applications so far are limited to photocatalytic degradation [21,22], radiosensitization [23] use in lithium batteries [24,25], and photoluminescence sensing [26,27].

This study aimed to investigate, for the first time, the efficacy of GeO_2_NPs in alleviating AD induced in the experimental animals. CeO_2_NPs were used for the comparison. To fulfill this goal, both nanoparticles were synthesized, fully characterized, and tested for their antioxidant activity. AD biochemical and molecular relevant markers of treated rats were quantified to fully understand the possible mechanism of action. Brain tissue pathology was also studied to confirm our hypothesis that GeO_2_NPs could protect brain cells from damage in an AD animal model and a set of possibly involved miRNAs were studied.

## 2. Materials and Methods

### 2.1. Chemicals

Germanium tetrachloride (GeCl_4_), 2,2-Diphenyl-1-picrylhydrazyl (DPPH), and (2,2′-azino-bis(3-ethylbenzothiazoline-6-sulfonic acid)) (ABTS) were purchased from Sigma Aldrich, St. Louis, MO, USA. Acetyl acetone (C_5_H_8_O_2_) and ethylene glycol (C_2_H_6_O_2_) were obtained from Alfa Aesar, Karlsruhe, Germany. Cerium nitrate hexahydrate (Ce(NO_3_)_3_.6H_2_O) with 99% purity was supplied from Chemlab, Zedelgem, Belgium. NaOH and AlCl_3_ were acquired from El-Gomhouria for Chemicals, Ramadan City, Egypt. All other reagents, solvents, and chemicals used for analysis met the quality criteria of the international standards.

### 2.2. Synthesis of Germanium Dioxide Nanoparticles (GeO_2_NPs)

GeO_2_NPs were synthesized as previously described [28] with some modifications. In brief, acetylacetone (10 mL) and ethylene glycol (10 mL) were properly mixed. Then, GeCl_4_ in a molar ratio of 1:4 to the mixture was added dropwise under magnetic stirring upon which a gel was formed, and 15 mL of distilled water was added to the reaction in a dropwise manner while stirring. The reaction was left for 4 h at 150 °C. The obtained white precipitate was collected by centrifugation and washed twice using distilled water. The obtained nanoparticles were then washed using ethanol and left in the oven at 50 °C overnight to dry. GeO_2_NPs powder was then calcinated in a furnace adjusted to 400 °C for 4 h.

### 2.3. Synthesis of Cerium Dioxide Nanoparticles (CeO_2_NPs)

CeO_2_NPs were synthesized using the precipitation method as described previously [29]. Briefly, 10 mL of 0.3 M NaOH was added dropwise to a stirring 10 mL of 0.1 M cerium nitrate hexahydrate Ce(NO_3_)_3_.6H2O. The reaction mixture was left stirring for 6 h in a water bath maintained at 70 °C. The formed precipitate was collected by centrifugation. At least three cycles of washing using distilled water were performed. The precipitate was washed once with absolute ethanol and left at 60 °C until completely dry. Annealing was conducted using a furnace at 400 °C for 4 h.

### 2.4. Characterization of GeO_2_NPs and CeO_2_NPs

X-ray powder diffraction (XRD) was conducted after annealing to verify phase purity according to the 2θ range of 10–80° with a scan rate of 8° min^−1^ (Philips X Pert diffractometer). Morphologically, they were characterized using high-resolution transmission electron microscopy (HR-TEM) using JEOL (JEM-2100, Osaka, Japan) at an accelerating voltage of 200 kV. Fourier transmission infrared (FTIR) spectroscopy was conducted (Model no. 4000, JASCO, Tokyo, Japan) with a range of 400–4000 cm^−^^1^ using KBr pellets. The surface charge was measured using the Malvern Zeta sizer Nano ZS instrument (Malvern Instruments, Malvern, UK). At least three measurements were performed [30].

### 2.5. Assessment of GeO_2_NPs and CeO_2_NPs Ex-Vivo Antioxidant Activity

The radial scavenging activity of the synthesized nanoparticles was quantified using both 2,2-Diphenyl-1-picrylhydrazyl (DPPH) and (2,2′-azino-bis(3-ethylbenzothiazoline-6-sulfonic acid)) (ABTS) assays. Vitamin C was the reference positive control in both assays. A serial dilution (10–100 µg/mL) of nanoparticles suspended in phosphate-buffered saline (PBS) was prepared by 15 min sonication in a water bath. In the DPPH assay, nanoparticle solution was mixed with an equal volume of DPPH solution (0.05 mg/mL) dissolved in methanol and the mixture was incubated in darkness for 2 h. The absorbance of the reaction mixture was measured at 517 nm using Shimadzu UV-2450 double beam spectrophotometer [30]. In the ABTS assay, radicals of ABTS were freshly prepared by mixing 7 mM of ABTS with 2.45 mM of potassium persulfate and incubating the mixture overnight in darkness. Nanoparticle solution was mixed with an equal volume of the prepared ABTS radical solution and absorbance was measured at 734 nm [31]. Measurements were conducted in triplicates and results were expressed as % of inhibition calculated according to Equation (1).

(1) $$Inhibition\left(\%\right)=\frac{Ac-As}{Ac}\times 100$$

where Ac was the absorbance of the working solution free of nanoparticles and As was the absorbance of the working solution mixed with the nanoparticles. From the plotted curves, the concentration needed to scavenge 50% of the produced radical (IC50) was determined [32].

### 2.6. Biological Experiment

Physiologically, estrogen levels change significantly during the menstrual cycle, as estrogen level elevates during the mid-follicular phase, then decrease after ovulation, followed by a second increase during mid-luteal phase, and subsequently decrease at the end of menstrual cycle. Since, estrogens have neuroprotective function against AD [3], this fluctuation in hormones level might influence the treatment in female experimental AD model. Apparently, due to the mentioned fact, and to avoid any bias associated with this hormonal imbalance, we established our AD-treated model in adult male Wistar albino rats according to the established protocols.

#### 2.6.1. Animals

Adult male *Wistar* albino rats weighing 200–220 g were procured from the Animal Care Unit at the National Research Centre, Cairo, Egypt. Food and water were available ad libitum. The room’s temperature was maintained at 20 ± 2 °C, and a 12:12 h light/dark cycle was sustained. The adaptation lasted at least one week before the initiation of the experimental protocol. Animal handling and management were following the Guidelines for the Animal Care and Use of the Ethical Committee for Medical Research of the National Research Centre, Egypt obtained in July 2021 with the approval number 1416072021.

#### 2.6.2. Assessment of NPs Toxicity and In Vivo Antioxidant Activity

Rats were randomly grouped into three groups (n = 4). The first group was the control that received PBS. The second group was the GeO_2_NPs group that received (37.5 mg/kg body weight) [33], and the third was the CeO_2_NPs group that received (0.5 mg/kg body weight) [34]. Treatment was administered as an intraperitoneal injection once weekly for 6 weeks. The vitality of rats was recorded, and rats were observed for signs of toxicity, such as behavioral changes, loss of weight, or excessive itching [35]. At the end of the treatment duration, rats were euthanized, and brain tissues were collected. The antioxidant whole brain homogenate levels of reduced glutathione (GSH) and superoxide dismutase (SOD) were measured using colorimetric activity assays [30]. Part of the tissues were paraformaldehyde fixed. The tissues were embedded in paraffin after dehydration. Sections were cut and mounted on glass slides. Immunohistochemical assessments of viability using Ki67 (Sigma Aldrich, USA) and the expression of Nrf2 (Santa Cruz Laboratory, Santa Cruz, CA, USA) were conducted [36]. Hematoxylin counterstaining was applied and the reactions sites of cerebellum, striatum, hippocampal dentate gyrus (DG), hippocampal prelimbic cortex (PL), and cerebral cortex were visualized as brown precipitates.

#### 2.6.3. Induction of AD

AD was induced via oral administration of AlCl_3_ dissolved in water (17 mg/kg body weight) for 6 weeks (5 days/week) [37].

#### 2.6.4. Experimental Protocol

Rats were randomly assigned into four groups (n = 10). Group 1 (AD + GeO_2_NPs): The AD group was treated with GeO_2_NPs dissolved in PBS that were intraperitoneally administered once weekly (37.5 mg/kg body weight) for 6 weeks [33]. Group 2 (AD + CeO_2_NPs): The AD group was treated with CeO_2_NPs dissolved in PBS intraperitoneally administered once weekly (0.5 mg/kg body weight) for 6 weeks [34]. Group 3 was an AD-induced group that was left untreated. Group 4 (control group) served as a negative control of healthy rats and was intraperitoneally injected with vehicle (PBS) once weekly for 6 weeks. Of note, the nanoparticles before administration were sonicated in a water bath for 10 min before injection to avoid aggregation phenomena due to the high temperatures reached during the calcination process. At the end of the experimental period, all rats were allowed to fast overnight, and blood samples were drained from the tail vein, following light anesthesia, to obtain sera samples. After centrifugation at 1800× *g* for 10 min at 4 °C, serum samples were cryopreserved in aliquots for analysis of biochemical markers. After blood collection, the rats were sacrificed, and brain tissues were harvested and thoroughly washed with PBS. The whole brain of a certain number of animals in each group was dissected and divided sagitally into two portions: The first portion was immediately collected in *RNAlater* RNA Stabilizing Reagent (Qiagen, Cat. No 76104, Germantown, MD, USA) and the homogenization of brain specimens was conducted. Then, total RNA, including miRNA, was extracted from the brain tissues of the four groups using Qiagen miRNeasy Kit (Qiagen, Catalog no. 217004) and stored at −80 °C for downstream molecular analysis. The second portion of brain tissues was weighed and immediately homogenized to give 10% (*w*/*v*) homogenate containing 50 mM Tris-HCl and 300 mM sucrose, pH 7.4 [38]. The brain homogenates were centrifuged at 1800× *g* for 10 min at 4 °C. A supernatant (10%) was stored in aliquots at −20 °C for the different biochemical determinations. Total protein concentration was also estimated to express the concentration of various biochemical parameters as mg^−^^1^ or µg^−^^1^ protein [39]. The whole brain of the remaining animals in each group was fixed in 10% formalin buffer for 24 h for the histological procedure.

#### 2.6.5. Biochemical Analyses (Serum and Brain)

Tau protein, phospho-tau protein, neurogranin (NG), and Aβ peptide 1-42 levels, and acetylcholinesterase (AchE) and monoamine oxidase (MAO) activities, were measured in serum and brain homogenate by enzyme-linked immunosorbent assay (ELISA) using kits purchased from Glory Science Co., Ltd., Del Rio, TX, USA according to operating instructions.

#### 2.6.6. Quantitative Analysis of microRNAs Expression

The purity and concentration of RNA were recorded using a NanoDrop spectrophotometer at the wavelength-dependent extinction coefficient 33 (NanoDrop Tech., Inc., Wilmington, DE, USA). Total RNA (10 ng) was converted to cDNA using Archive High-Capacity Reverse Transcription kit (Applied Biosystems, Waltham, MA, USA, Catalog no. 4368814) in T-Professional Basic Biometra PCR System (Biometra, Goettingen, Germany). For microRNA detection, specific forward primers for nine miRNAs (miRNA-9, miRNA-16, miRNA-29, miRNA-107, miRNA-125, miRNA-128, miRNA-137, miRNA-146, and miRNA-181) and a universal reverse primer 5′-GAACATGTCTGCGTATCTC-3′ were selected and retrieved from ORIGINE company (https://www.origene.com/catalog/ accessed on 5 November 2021, Table 1). Runs of qPCR were performed using Maxima^TM^ SYBR Green/ROX qPCR Master Mix (2X) (Thermo Fisher Scientific, Waltham, MA, USA, Catalog no. K0221), and beta-actin was used as an endogenous control. Appropriate negative and positive controls were also added. Real-time PCR reactions were carried out in StepOne™ Real-Time PCR System (Applied Biosystems) following the minimum information for publication of quantitative real-time PCR experiments (MIQE) guidelines. The results obtained were expressed in the quantification cycle (Cq), and the relative expression of each tested miRNA was assessed according to the formula (2^−ΔΔCt^) [40,41].

#### 2.6.7. Histopathological Procedure

Brain tissues were fixed in 10% buffered formalin for 24 h and washed with tap water. For dehydration, serial dilutions of ethyl alcohol were applied, after which, the specimens were cleared in xylene and processed using the paraffin wax embedding technique in a hot air oven at 56 °C for 6 h. Paraffin wax tissue blocks were sectioned using microtome at 4 microns thickness. Sections were collected on glass slides, deparaffinized, and stained for routine histological examination using hematoxylin and eosin (H&E) staining [36]. The histological sections were examined using an Olympus optical microscope (Tokyo, Japan).

### 2.7. Statistical Analysis

Statistical Package for the Social Sciences (SPSS) version 25 and Graph Pad Prism version 9.1.2 were used for analysis. Results are expressed as mean ± standard error of the mean (SE). One-way analysis of variance (ANOVA) was followed by the least significant difference (LSD) and Tukey tests for multiple comparisons. Two-sided *p* < 0.05 was considered statistically significant.

## 3. Results

### 3.1. Morphological Features of the Synthesized GeO_2_NPs and CeO_2_NPs and Their Ex-Vivo Antioxidant Activity

**Figure 1:**
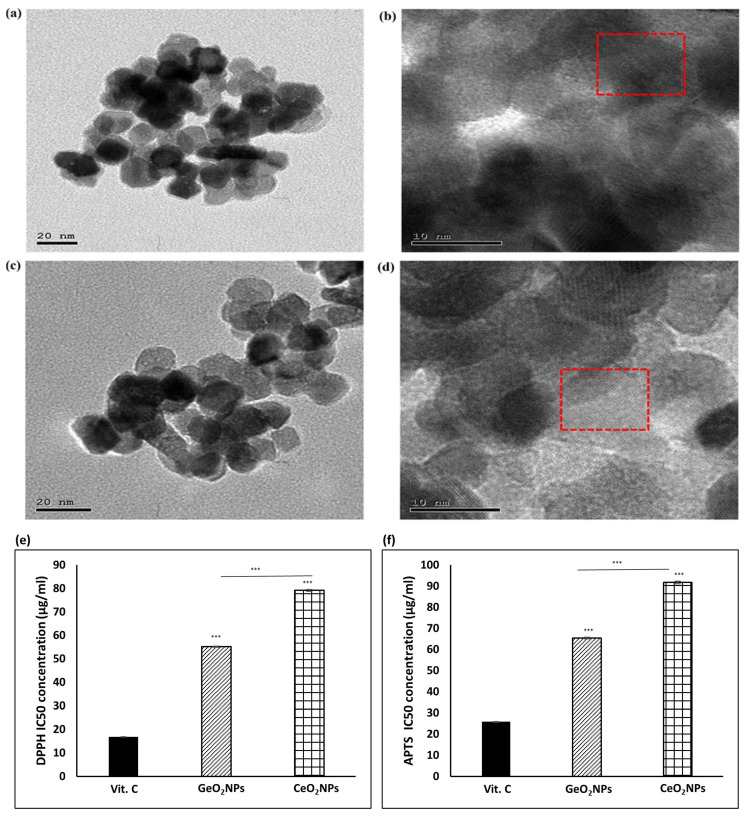
(**a**) High-resolution transmission electron microscope (HR-TEM) representative image of synthesized GeO_2_NPs, (**b**) a magnified HR-TEM image of GeO_2_NPs showing the lattice structure as indicated in the red rectangle, (**c**) HR-TEM representative image of synthesized CeO_2_NPs, (**d**) a magnified HR-TEM image of CeO_2_NPs showing the lattice structure as indicated in the red rectangle, (**e**) DPPH IC50 of GeO_2_NPs and CeO_2_NPs, (**f**) APTS IC50 of GeO_2_NPs and CeO_2_NPs. Vitamin C (Vit C) was used as a standard. (*n* = 3, *** depicts *p* ≤ 0.001).

The synthesized GeO_2_NPs and CeO_2_NPs were imaged using a high-resolution transmission electron microscope (HR-TEM) to examine their morphological appearance. As seen in (Figure 1a), GeO_2_NPs were quasi-spherical in shape with an average diameter of 20 nm. From the magnified HR-TEM image in (Figure 1b), the lattice structure of the nanoparticles was clearly detected, which confirmed their crystalline nature. The marked interplanar (d) spacing of 0.27 nm was for the plane (011), which was the prominent one. As shown in (Figure 1c), the synthesized CeO_2_NPs were 12–15 nm in diameter with a spherical shape. Typical metal oxide aggregation was observed. In the magnified HR-TEM (Figure 1d), the lattice structure of the nanoparticles was obvious. Based on this, a spacing of 0.29 nm was measured, which suggested that the synthesized CeO_2_NPs had a face-lefted cubic fluorite crystallographic structure.

Radical scavenging activity of GeO_2_NPs and CeO_2_NPs was confirmed by DPPH and APTS assays using vitamin C as a standard. According to the results of the DPPH assay (Supplementary Materials, Figure S1a), and APTS assay (Figure S1b), both nanoparticles exerted an antioxidant activity that was significantly weaker than vitamin C. Both nanoparticles showed a similar antioxidant activity when their concentrations were below 60 µg/mL. At higher concentrations, GeO_2_NPs activity was stronger than CeO_2_NPs. The concentrations needed to scavenge 50% of DPPH (DPPH IC50) of GeO_2_NPs was 55.1 ± 0.3 µg/mL while DPPH IC50 of CeO_2_NPs was 79.1 ± 0.4 µg/mL (Figure 1e). Similarly, APTS IC50 of GeO_2_NPs (65.4 ± 0.3 µg/mL) was lower than the respective one of CeO_2_NPs (91.7 ± 0.6) as seen in (Figure 1d).

### 3.2. XRD Pattern, FTIR, and Zeta Potential of the Synthesized GeO_2_NPs and CeO_2_NPs

**Figure 2:**
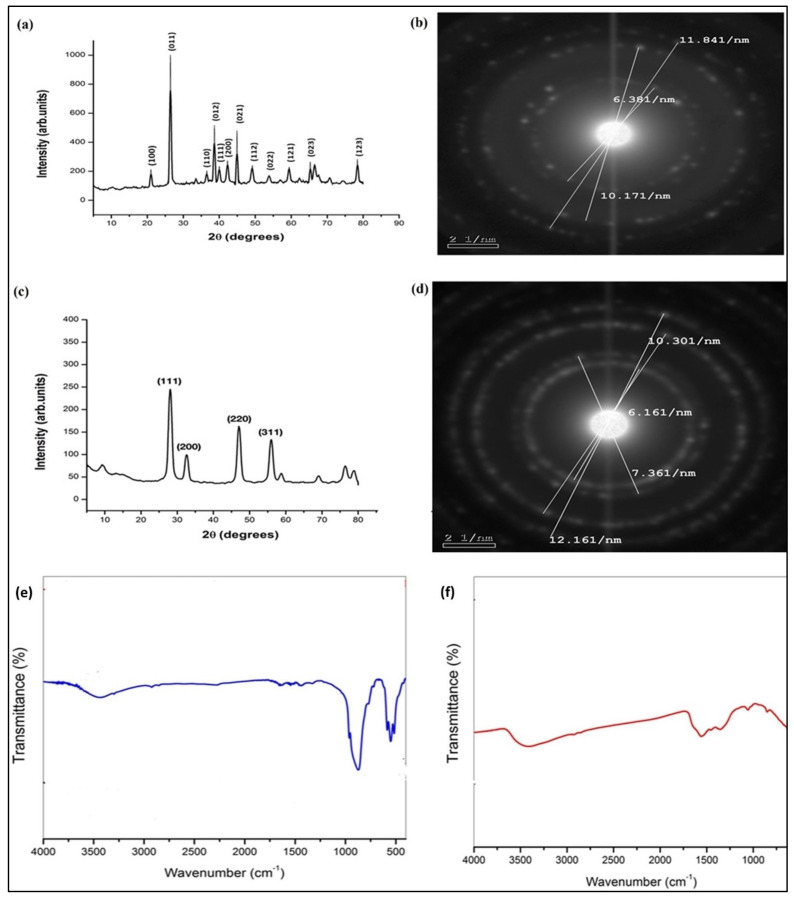
(**a**) GeO_2_NPs XRD pattern, (**b**) GeO_2_NPs selected area electron diffraction (SAED), (**c**) CeO_2_NPs XRD pattern, (**d**) CeO_2_NPs SAED, (**e**) FTIR spectrum of GeO_2_NPs, (**f**) FTIR spectrum of CeO_2_NPs.

The synthesized GeO_2_NPs were examined for their purity and crystallinity. The respective X-ray diffractogram (XRD) is shown in (Figure 2a). Results revealed that the synthesized nanoparticles were crystalline and show a pure hexagonal phase (space group: P32 2 1). The calculated cell parameters were a: 4.9113 A° and c: 5.6009 A°. There was a close matching with JCPDS No. 85-0473. The calculated average crystalline size applying the Scherrer equation was 24 nm. Selected area electron diffraction (SAED), shown in (Figure 2b), demonstrated the polycrystalline nature of the nanoparticles. By applying Bragg’s equation, the equivalent diffraction angles were 27°, 45°, and 53°. The XRD pattern of calcinated CeO_2_NPs is shown in (Figure 2c). The major high-intensity peaks were observed at 28°, 33°, 47°, and 56°, respectively, corresponding to the 111, 200, 220, and 311 crystal planes. Some minor peaks were also observed. The existence of those diffraction peaks matched the standard JCPDS data card (34-0394) for cerium oxide. The prepared CeO_2_NPs exhibited a pure face-lefted cubic polycrystalline structure as indicated by the sharp narrow and intense peaks. The calculated nanocrystalline particle size using the Debye Scherrer equation was 15 nm. SAED patterns presented in (Figure 2d) showed the polycrystalline nature of the nanoparticles. The corresponding interplanar spacing (d) was calculated following Bragg’s equation to be 0.325, 0.2717, 0.1941, and 0.1645 nm from the inner to the outer circle, respectively. They corresponded to 2θ 27.2, 32.5, 45.5, and 53.7°, respectively. Their respective miller indexations were 111, 200, 220, and 311. Thus, for both nanoparticles, the characteristic 2 theta degrees recorded in XRD analysis agreed with those calculated from the SAED pattern.

Both annealed GeO_2_NPs and CeO_2_NPs were examined by FTIR. The results for GeO_2_NPs are shown in (Figure 2e). The well-reported GeO_2_ characteristic bands [26,28] were observed. The first was the triplet band at 580, 550, and 510 cm^−1^, which corresponded to symmetric Ge-OH stretching, and the second was at 870 cm^−1^, which corresponded to GeO–Ge antisymmetric stretching. The band observed at 3500 to 3000 cm^−1^ was assigned to OH groups of adsorbed water molecules. The weak band at 1650 cm^−1^ was assigned to the bending vibration of water molecules and could also be assigned to –CO stretching vibration of carbonyl groups of the used organic solvents. FTIR results of CeO_2_NPs are shown in (Figure 2f). Peaks at 501 and 857 cm^−1^ were assigned to Ce-O bonds [42]. The signal at 1567 cm^−1^ was assigned to H-O-H bending vibration, which suggests that water molecules were adsorbed on the surface of the synthesized CeO_2_NPs [43]. A broad strong band at 3435 cm^−1^ was present. This band was assigned to the OH stretching of chemisorbed hydroxyl groups on the surface of the nanoparticles. Furthermore, the peaks at 1487 and 1367 cm^−1^ were assigned to νs (CO_3_) and δ (HO) modes of chemisorbed bicarbonate [44].

The zeta potentials of both nanoparticles were measured. GeO_2_NPs had an average surface charge of −27 ± 2 mV, while the surface charge of CeO_2_NPs was −17.5 ± 1.4 mV.

### 3.3. Safety of GeO_2_NPs and CeO_2_NPs and Their In Vivo Antioxidant Activity

**Figure 3:**
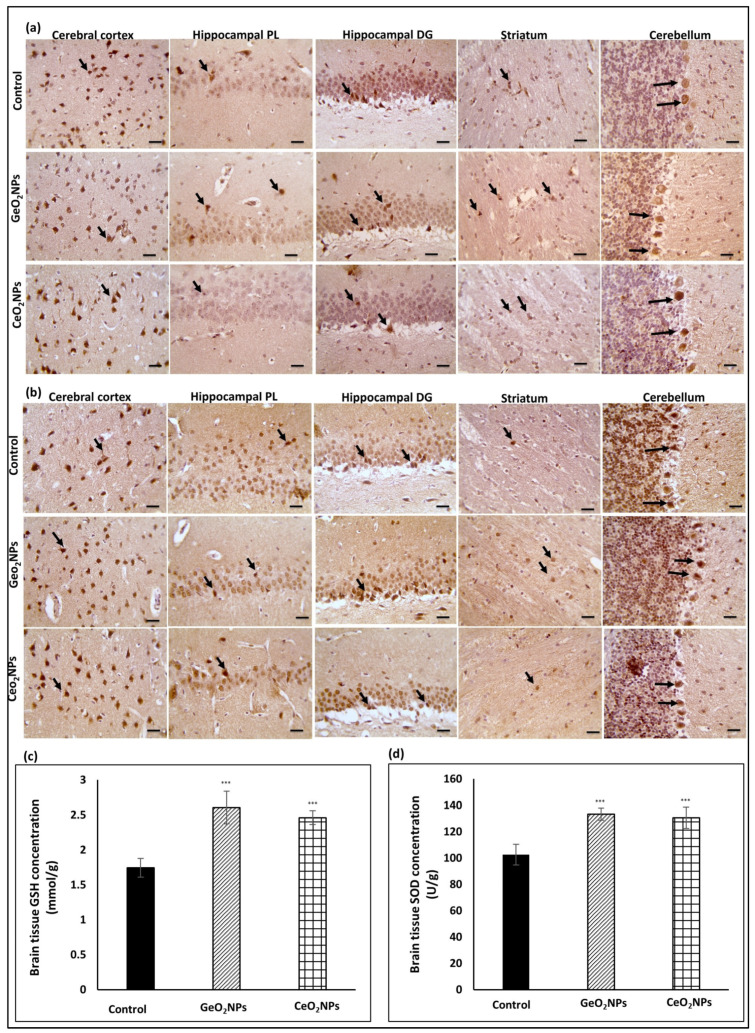
(**a**) Representative immunohistochemical photographs of control, GeO_2_NPs and CeO_2_NPs treated rats showing brain cerebellum, striatum, hippocampal dentate gyrus (DG), hippocampal prelimbic cortex (PL), and cerebral cortex stained with Ki67, (**b**) stained with Nrf2 antibody. Magnification was 400× and bar is 50 µm. Black arrows depicted positive regions for the applied antibodies, (**c**) brain tissue concentration of GSH, (**d**) brain tissue concentration of SOD. Experiments were repeated three times, presented are average and standard deviation, *** indicated *p* value ˂ 0.001.

During the entire treatment duration that lasted for 6 weeks, treated rats did not show mortality or signs of abnormal behavior that might indicate toxicity. The brain section of both GeO_2_NPs and CeO_2_NPs treated rats were positive to Ki67 (Figure 3a) confirming the safety of the selected doses. Tissue sections were positive to Nrf2 (Figure 3b) and tissue levels of GSH (Figure 3c) and SOD (Figure 3d) were significantly higher in treated groups than in the control (*p* ˂ 0.05).

### 3.4. Serum Biochemical AD-Relevant Markers in Response to Treatments

Upon administration of AlCl_3_ in the AD group, the serum levels of tau protein and phosphorylated tau were 2-fold and 3.6-fold higher than the control group, respectively, while the serum level of neurogranin (NG) declined to half of the control, confirming the success of the AD model (Table 2). Treating AD-bearing rats with CeO_2_NPs resulted in significant depletion (*p* ˂ 0.05) in serum tau protein and phosphorylated tau accompanied by significant elevation (*p* ˂ 0.05) in the NG serum level versus the AD-bearing rats, indicating their therapeutic efficacy. On the other hand, the AD-bearing rats treated with GeO_2_NPs had tau protein and NG levels similar to the control group and phosphorylated tau markedly lower than the group treated with CeO_2_NPs.

In the AD group, the activities of serum acetylcholinesterase (AchE) and monoamine oxidase (MAO) were 3-fold and 2-fold higher than in the control group, respectively, whereas serum levels of Aβ peptide 1-42 were reduced by half (Table 2). Treating AD-bearing rats with CeO_2_NPs significantly (*p* ˂ 0.05) reduced AchE and MAO activities in contrast to the AD-bearing rats, but their values were still higher than controls. On the other hand, treating AD-bearing rats with GeO_2_NPs reduced serum AchE and MAO activities remarkably less than those in CeO_2_NPs-treated rats, where the values were similar to controls. In both treatment groups (AD + CeO_2_NPs and AD + GeO_2_NPs), serum Aβ peptide 1-42 level significantly increased (*p* ˂ 0.05) compared to the AD-bearing group, but it was higher in the GeO_2_NPs-treated group than the CeO_2_NPs-treated group.

### 3.5. Brain Biochemical AD-Relevant Markers in Response to Treatments

Brain tau protein and phosphorylated tau levels were 2-fold and 2.7-fold higher in the AD group relative to controls, respectively, while brain NG level was significantly diminished (*p* ˂ 0.05) in the AD group versus the control group. These biomarkers were restored almost to control levels in the AD-bearing rats treated with GeO_2_NPs. In this regard, GeO_2_NPs showed a more prominent effect than CeO_2_NPs (Table 3).

Brain Aβ peptide level, AchE, and MAO activities showed significant elevation (*p* ˂ 0.05) in AD-bearing rats when compared with the control counterparts. Treatment of AD-bearing rats with either CeO_2_NPs or GeO_2_NPs brought about significant regression (*p* ˂ 0.05) in these markers in comparison with AD-bearing rats. Of note, GeO_2_NPs displayed a more pronounced impact than CeO_2_NPs regarding these parameters (Table 3).

### 3.6. Histopathological Observations

**Figure 4:**
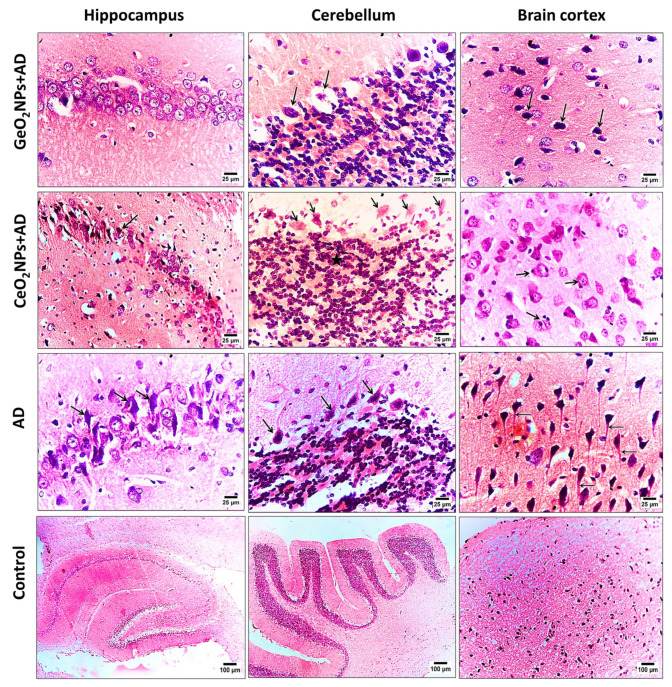
Photomicrograph of H&E-stained hippocampus, cerebellum, and brain cortex sections of AD-induced rats treated with GeO_2_NPs (AD + GeO_2_NPs), treated with CeO_2_NPs (AD + CeO_2_NPs), left untreated (AD) and healthy rats (Control). Arrows depicted degeneration and necrosis of neurons and arrows in the brain section of AD group depicted diffused neurofibrillary tangles. The star in the cerebellum section of CeO_2_NPs + AD group depicts where the cells of granular area had gliosis, degeneration, and clumps together. Magnification was 400× in all samples except in the sections of the control where it was 40×.

Brain sections of the rat groups were stained with H&E and examined for their histopathological features. In the four groups, the hippocampus, cerebellum, and brain cortex were investigated. The (Figure 4). The AD group treated with GeO_2_NPs was the only one showing a normal feature of pyramidal cells and neurons of the hippocampus whereas the AD treated group with CeO_2_NPs showed advanced degeneration and necrosis of neurons and pyramidal cells. The degradation and necrosis were aggravated and diffused in the AD group. Cerebellum of GeO_2_NPs and CeO_2_NPs treated groups showed degeneration and necrobiosis of Purkinje cells. Furthermore, cells of the granular area showed gliosis and degeneration and clump together. However, it is worth mentioning that those histopathological features were milder than the observed ones in the AD group. The brain cortex of GeO_2_NPs and CeO_2_NPs treated AD-induced rats showed necrosis of neurons. The recovery process and immune response were evident by the observed proliferation of astrocyte and microglial cells. The AD group showed necrobiosis of neurons and diffused neurofibrillary tangles. The control group showed normal histopathological features.

### 3.7. MicroRNAs Transcriptomic Signature

**Figure 5:**
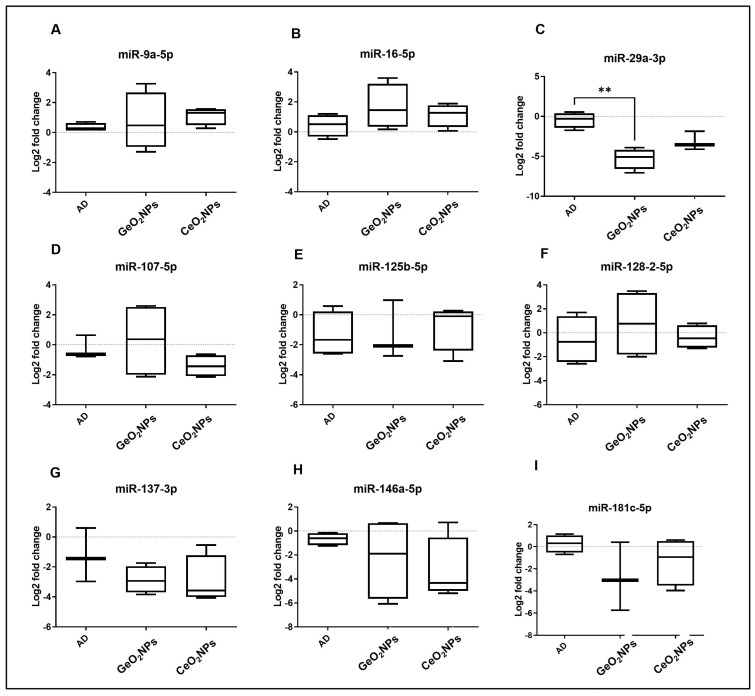
MicroRNA quantification in brain tissues of rats; AD, GeO_2_NPs-treated group, and CeO_2_NPs-treated group. The logarithmic transformation value of log 2-fold changes is shown. One-way ANOVA followed by post hoc Tukey tests were used. ** *p* < 0.01.

A literature search and bioinformatics analysis using mirPath v.3 KEGG reverse search (http://snf-515788.vm.okeanos.grnet.gr/) accessed on 5 November 2021 for AD KEGG pathway (rno05010) revealed nine putative AD-related microRNAs: rno-miR-9a-5p, rno-miR-16-5p, rno-miR-29a-3p, rno-miR-107-5p, rno-miR-125b-5p, rno-miR-128-2-5p, rno-miR-137-3p, rno-miR-146a-5p, and rno-miR-181c-5p. Using real-time PCR, we assessed the expression of these nine miRNAs in brain tissue from treated and non-treated AD groups (Figure 5). There was no significant difference in microRNAs expression between AD and control animals; however, miR-29a-3p was significantly down-regulated in the GeO2NPs–treated group compared to the AD group (*p* = 0.0080).

## 4. Discussion

The main purpose of this study was to assess for the first time the potential therapeutic effect of GeO_2_NPs compared to CeO_2_NPs in an experimental model of AD. GeO_2_NPs and CeO_2_NPs were synthesized using a facile co-precipitation synthetic method. Unlike other methods, co-precipitation can be easily optimized. Adjusting the temperature, reaction time, and the ratio between acetylacetone and germanium tetrachloride could alter the size of the produced GeO_2_NPs [28].

Similarly, for the synthesis of CeO_2_NPs, numerous methods have been reported. One is the sonochemical method [45]. In this study, the precipitation method was selected since it is facile and easily scalable, unlike others requiring high pressure or temperature, or capping agents. In the precipitation reaction, upon the slow addition of a strong alkali (in our case NaOH), the reaction pH increased to 10, and a pinkish-white precipitate was formed. This precipitate could be easily collected by centrifugation, and the color turned yellowish upon drying.

Both nanoparticles were annealed at 400 °C for 4 h. The selected annealing temperature was proven not to alter the particle size or the crystalline nature of the formed nanoparticles [46]. GeO_2_NPs were quasi-spherical in shape with a diameter of 20 nm. In contrast to the previous study of Nejaty-Moghadam and his colleagues [28], we changed the reaction duration from 30 min to 4 h, and the reaction temperature was raised to 150 °C instead of 50 °C while maintaining a 4:1 ratio between acetylacetone and germanium tetrachloride. In the case of CeO_2_NPs, the produced nanoparticles were 12–15 nm in diameter, as reported earlier [47] with a spherical shape.

The nanoparticles were crystalline and GeO_2_NPs showed a pure hexagonal phase. This alpha quartz-like hexagonal structure is known for its stability at high temperatures and pressure [48]. The obtained XRD pattern is in line with previous studies [19,28]. CeO_2_NPs showed a face-lefted cubic phase. Based on oxygen niche, there are various phases of CeO_2_NPs, including fluorite, bixbyite, rhombohedral, and triclinic phases [49]. Based on XRD results, our synthesized CeO_2_NPs had a pure fluorite phase. This phase structure allows for redox cycles and this explains the prominent antioxidant activity of CeO_2_NPs [50]. The fabrication of both nanoparticles was confirmed by FTIR analysis, where characteristic absorption peaks for GeO_2_NPs at 870 cm^−1^ and CeO_2_NPs at 857 cm^−1^ were present. Chemisorbed molecules on the surface, such as water and bicarbonate, were also observed. They provide the hydroxyl groups through which fabricated nanoparticles can interact with other molecules, such as carbohydrates [51].

The surface charge of the nanoparticles performs a vital role in their pharmacokinetic profile. The overall charge determines the interaction with carrier proteins (e.g., albumin), cellular uptake, and subcellular distribution [52]. GeO_2_NPs zeta potential was −27 mV while it was −17 mV for CeO_2_NPs as an average surface charge. Bearing a negative charge increases the repulsive forces between the nanoparticles and fosters the formation of stable colloidal suspensions [53].

Many studies showed that AD is linked with oxidative stress. Generated radicals cause lipid peroxidation, protein oxidation, DNA damage, and glycoxidation within the brain tissues. Deficiency of antioxidants and prolonged oxidative stress cause neurodegeneration [54]. Accordingly, antioxidants, such as vitamin C, and vitamin E, are strongly recommended as AD therapies [55]. CeO_2_NPs are well reported for their antioxidant activity and redox behavior, which explained the obtained results of our conducted DPPH and APTS assays [56]. It was reported before that organogermanium could protect human dermal cells from oxidative-stress-induced cell death through the regulation of cell death and inflammatory genes [57] and its administration elevated the antioxidant levels of treated rats [58]. Our study showed that GeO_2_NPs exerted radical scavenging activity in a dose-dependent manner and their activity was stronger than CeO_2_NPs at concentrations above 60 µg/mL. This finding was confirmed by both DPPH and APTS assays and it is worth mentioning that this is the first report on this activity.

The doses of GeO_2_NPs and CeO_2_NPs administered to the rats were proven to be safe. They have improved the redox status of the animals as indicated by the significant increase in the GSH and SOD levels compared to the control. This might be attributed to the activation of Nrf-2 pathway. While AD cannot be cured, boosting the brain antioxidant status is desirable and reported as a prophylactic practice to AD and other neurodegenerative diseases [59].

In the present research, the AD model was established and treated with either CeO_2_NPs or GeO_2_NPs. Treatment efficiency was assessed by measuring the serum and tissue tau protein, phosphorylated tau, neurogranin (NG), Aβ peptide 1-42, Acetylcholinesterase (AchE), and monoamine oxidase (MAO). Our findings indicated that GeO_2_NPs were superior to CeO_2_NPs since they restored biomarker levels almost to the control values. Those findings agreed with their radical scavenging activity results and thus might be related to their antioxidant activity.

A variety of new biomarkers for early diagnosis of AD have emerged in recent years. The level of neurogranin (NG) in cerebrospinal fluid (CSF) showed a positive correlation with total tau and phosphorylated tau protein in CSF in AD patients. Tau is the main neuronal microtubule protein in the brain of AD patients; it is abnormally hyperphosphorylated and aggregated into paired helical filaments, which appear as neurofibrillary tangles (NFTs). This suggests that tau protein may be a promising target for developing therapeutic medications for AD [60].

Tau primarily functions by stimulating microtubule assembly and stabilizing the microtubule structure and its phosphorylation state regulates this function. Normal brain tau contains two to three moles of phosphate per mole [61]. In AD brains, Tau is abnormally hyperphosphorylated and the result is the major subunit of paired helical filaments (PHFs) and straight filaments (SFs), which form NFTs, neuropil threads, and plaque dystrophic neurites. Tau hyperphosphorylation appears to result in neurofibrillary degeneration in AD [60]. These results strongly support our findings regarding tau and phospho tau levels.

It has been shown that the number of NFTs, not the presence of plaques, correlates most closely with the degree and/or presence of dementia in AD. It appears that neurofibrillary degeneration is necessary for the clinical manifestation of the disease. Depending on the molecular mechanism of tau-mediated neurofibrillary degeneration, therapeutic strategies may include increased tau clearance, inhibition of abnormal tau hyperphosphorylation, and suppression of tau misfolding/aggregation [60].

Neurogranin (NG) is a post-synaptic protein that is especially abundant in the dendritic spines of the hippocampus, amygdala, caudate, and putamen. It binds the calcium-binding protein calmodulin (CaM) to modulate calcium signaling and synaptic plasticity [62]. NG has a vital role in the initiation of long-term potentiation (LTP) in the hippocampus. In the normal human brain, NG expression is the highest in associative cortical areas, while in AD, NG levels are depleted in the cortex and hippocampus, reflecting synaptic loss [63]. Studies of blood NG are limited, and they have not described significant variations between AD and controls [64].

In animal models, NG knockdown restrains synaptic LTP and affects mental capacity, while NG up-regulation advances LTP and further develops cognition. Comparative investigations are accounted for in patients with AD, as NG is depleted in the brain and this exhaustion is related to more unfortunate mental performance. NG can identify the neurotic changes of AD at prior stages, even in the mild cognitive impairment (MCI) stage, and subsequently is viewed as a potential biomarker for the determination of AD [65].

The level of NG in blood plasma exosomes of patients with AD and MCI was low compared to controls, and NG levels in blood plasma exosomes of patients with AD and MCI-AD were also lower than those in patients with MCI. These findings emphasized the clinical proof that NG in blood exosomes can be used as a cognitive biomarker for AD and MCI [66]. Kvartsberg et al. [67] reported that the level of NG in the blood plasma exosomes was the same as that of AD patient brain tissues, but the level in the CSF was the reverse. Liu et al. [66] hypothesized that the raise of NG level in CSF and the decline of NG in blood plasma exosomes and brain tissues in patients with AD and MCI-AD were related to the decomposition of NG into many peptides modified by disulfide bridges or glutathione and released into CSF.

Unlike the biochemical pathway of Aβ and tau, NG acts in response to synaptic loss or synaptic plasticity disorder. Synaptic dysfunction and deterioration are believed to be central issues in AD pathology from early stages because learning and memory are formed through synaptic plasticity [68]. This evidence reveals that NG can be an encouraging factor for the early identification of cognitive deterioration. NG binding to CaM weakens when the synaptic structure is interrupted, affecting the diffusion of Ca^2+^ between the synapses and the formation of LTP, leading to early cognitive decline. More significantly, studies in humans have shown that expression of NG declined in the frontal and parietal cortices of AD patients, and NG was significantly correlated with the degree of amyloid and tau pathology [66].

Acetylcholinesterase (AchE) is a catalytic enzyme responsible for the breakdown of the acetylcholine. Since AD can be regarded as a loss of cholinergic neurons, reducing AchE is a reasonable modality. Additionally, AchE is known to act as an amyloidogenic factor supporting the deposition of tau protein and augmenting its correlated neurotoxicity [69].

Monoamines assume a huge part in cognition managing memory and learning. Abnormal monoaminergic signaling generally goes with cholinergic deficiencies, which supports early phase changes in monoaminergic tone, compounded by cholinergic deficits, as contributing elements to the mental deterioration in AD [70]. Specific changes in monoaminergic capacity could be a significant contributor to neuropsychiatric symptoms, including depression, irritability, aggressive outbursts, and delusions [71]. Notably, these diseases reflect region-specific noradrenergic and serotoninergic insults; in this way, MAO-A and MAO-B dysfunction, and a monoaminergic injury, could participate to a spectrum of symptoms and pathology in both early and sustained AD development [70].

MAO may also be a factor in neuropathology because of the generation of hydrogen peroxide as a by-product of the deamination reaction. The ensuing oxidative stress and potential for cell death—invariably involving the mitochondria—would be exacerbated when antioxidant systems are compromised as in the case of AD [72]. AD subjects usually show an increase in MAO-B in the brain and platelets. Studies demonstrated an increased binding of the MAO-B-specific ligand 11C-deuterium-L-deprenyl (11C-DED) early in pre-symptomatic Familial Alzheimer’s Disease (FAD) cases, which was thought to boost astrocytosis. Nevertheless, the mechanism by which MAO-B affects AD pathogenesis is not known. Some initial outcomes suggest that MAO-B is correlated with γ-secretase activity [73].

In the present study, CeO_2_NPs and GeO_2_NPs significantly improved tau and phospho tau protein through their antioxidant effect. Dowding et al. [14] stated that 2.9 nm citrate/EDTA-stabilized CeO_2_NPs administered intravenously to healthy rats seem to cross the intact blood- brain barrier (BBB). By contrast, another study by Kim et al. [74] used phospholipid-polyethylene glycol-coated CeO_2_NPs, yet these particles were found only in low amounts in normal brain tissue. In AD cases, BBB is dysfunctional, thus nanoparticles are more likely to adequately present within brain tissues [75].

In vitro and in vivo, CeO_2_NPs demonstrated antioxidant properties due to their superoxide dismutase (SOD) and catalase (CAT)-like activities. In general, CeO_2_NPs remove ROS due to surface self-regeneration [76]. However, the excessive generation of ROS found in neurodegenerative diseases can harm cells, necessitating exogenous aids. Considering this, antioxidants are frequently used. On the other hand, antioxidants are unable to cross the BBB, emphasizing the importance of nanoparticle-based antioxidant delivery systems [77]. CeO_2_NPs, which continually scavenge ROS, are of such delivery systems.

CeO_2_NPs have been proposed by Naz et al. [15] as a potential treatment candidate for mitochondrial injury. When compared to other ROS modulators, CeO_2_NP’s unique redox behavior makes it a strong antioxidant. CeO_2_NPs can modify the brain-derived neurotrophic factor signal transduction pathway in AD, retarding the apoptotic effect of the disease in neuronal cells. When conjugated with metal chelators or polyethylene glycol coatings, they can reduce Aβ aggregation [78]. Studies have also shown that CeO_2_NPs inhibited Aβ-mediated mitochondrial fragmentation via a mechanism involving the decrease in DRP1 S616 hyperphosphorylation [14]. Although it is doubtful that nitrosative stress causes mitochondrial breakage and neuronal cell death by phosphorylating a single protein target, such as DRP1, these findings describe one possible mechanism by which CeO_2_NPs can mitigate the downstream effects of RNS/ROS. Before moving on to human therapeutic trials, more in vitro and in vivo investigations are needed.

On the molecular level, miRNAs have frequently been found to be disorganized in AD. Many miRNAs have been identified as important factors in the regulation of cognitive functioning and memory processes lost in AD [79]. As a result, distinct miRNAs may perform a protective or promoting function in AD. In the current study, the expression of nine miRNAs was investigated in brain tissue. Among the tested miRNAs, miR-29a-3p, a well-known brain-enriched miRNA, is considered to be a potential biomarker for AD, especially from CSF, as it is significantly increased in AD patients compared to controls. In vitro, it was evident that miR-29a-3p can regulate beta-site APP-cleaving enzyme (BACE) expression. Moreover, miR-29a-3p has potentially been involved in the up-regulation of amyloid precursor protein and BACE1 expression. In contrast, Hébert and his team [6] stated that the miR-29a is significantly decreased in AD patients displaying abnormally high BACE1 protein. It is not clear whether this correlation of miR-29a-3p/BACE expression is restricted to certain areas of the brain or broadly expressed. In the current investigation, miR-29a-3p was shown to indicate a successful therapeutic response using GeO_2_NPs.

## 5. Conclusions

Based on the gathered data, GeO_2_NPs and CeO_2_NPs are emerging as promising new tools to be authenticated as possible pharmacological treatments in neurodegenerative diseases, especially AD. The validation of new therapeutic agents suggests the understanding of their mechanisms of action; therefore, we report the effects of GeO_2_NPs and CeO_2_NPs on tau and phospho tau proteins, Aβ peptide 1-42, and neurogranin, acetylcholinesterase, and monoamine oxidase, all known to be engaged in neuronal survival. The results obtained strongly support a neurotrophic role for these nanoparticles and indicate their use as a modulator of pathways crucial for neuronal survival.

## Figures and Tables

**Table 1:** Primer sequences of the analyzed microRNAs. List of forward primers for the analyzed microRNAs.

microRNAs	The Sequence of Forwarding Primers
rno-miR-9a-5p	5′-TCTTTGGTTATCTAGCTGTAT-3′
rno-miR-16-5p	5′-TAGCAGCACGTAAATATTGG-3′
rno-miR-29a-3p	5′-ACTGATTTCTTTTGGTGTTC-3′
rno-miR-107-5p	5′-AGCTTCTTTACAGTGTTGCCT-3′
rno-miR-125b-5p	5′-TCCCTGAGACCCTAACTTGT-3′
rno-miR-128-2-5p	5′-GGGGGCCGATGCACTGTAA-3′
rno-miR-137-3p	5′-ACGGGTATTCTTGGGGTGGAT-3′
rno-miR-146a-5p	5′-TGAGAACTGAATTCCATGGG-3′
rno-miR-181c-5p	5′-CATTCAACCTGTCGGTGA-3′

**Table 2:** Measured biomarkers in serum. Serum tau, phospho-tau protein, neurogranin (NG), Aβ peptide 1-42, acetylcholinesterase (AchE), and Monoamine oxidase (MAO) in the experimental groups (Mean ± SE).

Marker	AD + GeO_2_NPs	AD + CeO_2_NPs	AD	Control
Tau protein pg/mL	280 ± 9 *	348 ± 8 *	449 ± 8 *	220 ± 9
Phospho Tau pg/mL	75 ± 3	108 ± 5 *	171 ± 10 *	48 ± 5
Neurogranin (NG) ng/mL	1.22 ± 0.05	0.99 ± 0.04 *	0.67 ± 0.04 *	1.40 ± 0.05
Aβ peptide 1-42 pg/mL	14.9 ± 0.5 *	13.3 ± 0.4 *	11.5 ± 0.4 *	20.3 ± 0.7
Acetylcholinesterase (AchE) ng/mL	198 ± 4 *	253 ± 2 *	313 ± 3 *	113 ± 3
Monoamine oxidase (MAO) pg/mL	1868 ± 40 *	2796 ± 37 *	3372 ± 32 *	1815 ± 23

* Significant change at *p* > 0.05 in comparison with control and/or AD-induced group.

**Table 3:** Measured biomarkers in tissue. Brain tau, phospho-tau protein, neurogranin (NG), Aβ peptide 1-42, acetylcholinesterase (AchE), and Monoamine oxidase (MAO) in the experimental groups (Mean ± SE).

Marker	AD + GeO_2_NPs	AD + CeO_2_NPs	AD	Control
Tau protein ng/µg protein	17.7 ± 0.3	21.5 ± 0.4 *	26.6 ± 0.7 *	14.6 ± 0.5
Phospho -Tau pg/µg protein	185 ± 4	264 ± 2 *	380 ± 4 *	140 ± 3
Neurogranin (NG) ng/mg protein	470 ± 7	419 ± 3 *	368 ± 2 *	535 ± 5
Aβ peptide 1-42 ng/µg protein	10.6 ± 0.3 *	14.3 ± 0.2 *	16.9 ± 0.2 *	7.5 ± 0.4
Acetylcholinesterase (AchE) U/mg protein	595 ± 9 *	705 ± 4 *	790 ± 2 *	553 ± 4
Monoamine oxidase (MAO) nmol/mg protein	60.7 ± 0.7 *	68.3 ± 0.7 *	81.4 ± 1.0 *	52.4 ± 1.4

* Significant change at *p* > 0.05 in comparison with control and/or AD-induced group.

## Data Availability

Data are available upon request from the corresponding author.

## Supplementary Materials

The following supporting information can be downloaded at: https://www.mdpi.com/article/10.3390/pharmaceutics15051386/s1, Figure S1: (a) antioxidant activity of GeO_2_NPs and CeO_2_NPs tested by DPPH assay, (b) antioxidant activity of GeO_2_NPs and CeO_2_NPs tested by APTS assay. Vitamin C (Vit. C) was used as a standard.
