# Supplementary material for: Germanium Dioxide Nanoparticles Mitigate Biochemical and Molecular Changes Characterizing Alzheimer’s Disease in Rats
Source: Pharmaceutics. 2023 Apr 30;15(5):1386. doi: 10.3390/pharmaceutics15051386 (PMC10220875; doi:10.3390/pharmaceutics15051386)
Supplement: Supplementary file 1 [file pharmaceutics-15-01386-s001.zip › pharmaceutics-2122002-supplementary.pdf]

pharmaceutics

MDPI

Supplementary Materials: Germanium Dioxide Nanoparticles mitigate Biochemical and Molecular Changes Characterizing Alzheimer's Disease in Rats

Sara A. Abdel Gaber, Amal H. Hamza, Mohamed A. Tantawy and Eman A. Toraih,Hanaa H. Ahmed

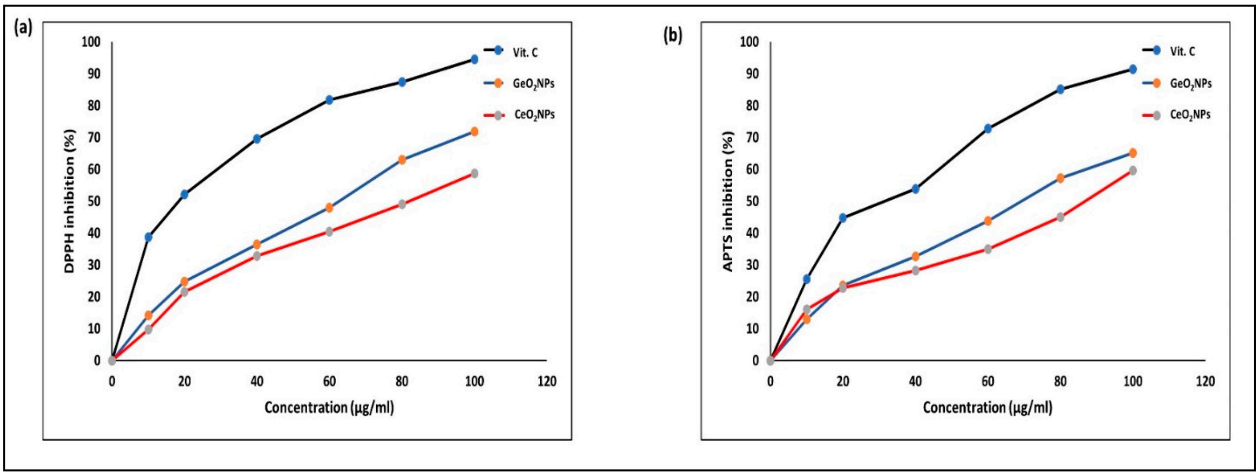

Figure S1. (a) antioxidant activity of GeO_2NPs and CeO_2NPs tested by DPPH assay, (b) antioxidant activity of GeO_2NPs and CeO_2NPs tested by APTS assay. Vitamin C (Vit. C) was used as a standard.
